# Temsirolimus Inhibits Proliferation and Migration in Retinal Pigment Epithelial and Endothelial Cells via mTOR Inhibition and Decreases VEGF and PDGF Expression

**DOI:** 10.1371/journal.pone.0088203

**Published:** 2014-02-26

**Authors:** Raffael Liegl, Susanna Koenig, Jakob Siedlecki, Christos Haritoglou, Anselm Kampik, Marcus Kernt

**Affiliations:** Department of Ophthalmology, Ludwig-Maximilians-University, Munich, Germany; Eye Hospital, Charité, Germany

## Abstract

Due to their high prevalence, retinal vascular diseases including age related macular degeneration (AMD), retinal vein occlusions (RVO), diabetic retinopathy (DR) and diabetic macular edema have been major therapeutic targets over the last years. The pathogenesis of these diseases is complex and yet not fully understood. However, increased proliferation, migration and angiogenesis are characteristic cellular features in almost every retinal vascular disease. The introduction of vascular endothelial growth factor (VEGF) binding intravitreal treatment strategies has led to great advances in the therapy of these diseases. While the predominant part of affected patients benefits from the specific binding of VEGF by administering an anti-VEGF antibody into the vitreous cavity, a small number of non-responders exist and alternative or additional therapeutic strategies should therefore be evaluated. The mammalian target of rapamycin (mTOR) is a central signaling pathway that eventually triggers up-regulation of cellular proliferation, migration and survival and has been identified to play a key role in angiogenesis. In the present study we were able to show that both retinal pigment epithelial (RPE) cells as wells as human umbilical vein endothelial cells (HUVEC) are inhibited in proliferating and migrating after treatment with temsirolimus in non-toxic concentrations. Previous studies suggest that the production of VEGF, platelet derived growth factor (PDGF) and other important cytokines is not only triggered by hypoxia but also by mTOR itself. Our results indicate that temsirolimus decreases VEGF and PDGF expression on RNA and protein levels significantly. We therefore believe that the mTOR inhibitor temsirolimus might be a promising drug in the future and it seems worthwhile to evaluate complementary therapeutic effects with anti-VEGF drugs for patients not profiting from mono anti-VEGF therapy alone.

## Introduction

Age related macular degeneration (AMD), macular edema (ME) following retinal vein occlusions (RVO) and diabetic macular edema (DME) as a complication of diabetic retinopathy (DR) are major reasons for severe vision loss and legal blindness in the western world [1]–[3]. With an expected increase in patients suffering from diabetes mellitus as well as the rising mean age of the population over the next decades even more patients will be affected in the future resulting in a tremendous socio-economic burden [4].

The pathogenesis of retinal vascular disease is complex and yet not fully understood [5], [6]. However, cellular proliferation and vascular leakage are found in AMD, ME following RVO, as well as in DME, resulting in pathologic fluid accumulation in the macular area. Moreover is (sub)retinal neovascularization in AMD a severe complication and evidence exists, that this event is mainly triggered by proliferation and migration of endothelial and retinal pigment epithelial cells [7]. In addition, it is well known that besides a mechanical defect in different structures, such as the endothelium in retinal vessels and the outer blood retinal barrier formed by the retinal pigment epithelium and Bruchs membrane, a common underlying mechanism includes the increased production of angiogenic and inflammatory components due to increased hypoxic retinal conditions [8]–[11]. Although it is sufficiently clear, that the three diseases are different in their development and pathological sequence, it has been shown, that hypoxia and the deregulation of a large number of various growth factors such as platelet derived growth factor (PDGF), placenta derived growth factor (PlGF), connective tissue growth factor (CTGF) and particularly vascular endothelial growth factor (VEGF) play a crucial role in their etiopathologies [12]–[15]. Based on this knowledge selective intravitreal inhibition of VEGF has become a safe and effective primary treatment approach in the therapy of neovascular AMD, ME following RVO, and DME, as well as in several other ocular conditions that are characterized by macular edema. Today, the intravitreal injection of VEGF antibodies such as bevacizumab and ranibizumab or fusion proteins such as aflibercept [16], [17] have therefore become common clinical practice. Large studies (e.g. RESOLVE, MARINA or VIEW) could clearly show that a significant number of patients suffering from macular edema improved in terms of edema resolution as well as visual acuity [18]–[20]. However, a number of patients do not improve from this treatment and for these cases alternative treatment options should be investigated.

Hypoxia plays a central role in the development and progression of retinal vascular diseases. A number of studies could clearly link hypoxia with several retinal diseases characterized by retinal ischemia and subsequent pathological angiogenesis involving the up-regulation of VEGF and other VEGF-related polypeptides such as PlGF [21]–[23]. The binding of these VEGF-family proteins to their receptor tyrosine kinases VEGFR-1, VEGFR-2 and VEGFR-3 is followed by numerous downstream effects leading to proliferation, cell migration, vascular permeability and endothelial inflammation. The most angiogenic effect of VEGF *in-vivo* is linked with its binding to VEGFR-2 [24]. One of the most important signaling pathways among several downstream of VEGFR-2 is PI3K/Akt [25], [26] which subsequently activates the mammalian target of Rapamycin (mTOR) [27]. mTOR is a serine-threonine protein kinase that plays an important role in signal transduction pathways that control cell growth and angiogenesis and has been a target to many cancer therapy approaches *in-vitro* and *in-vivo* [28], [29]. Temsirolimus is an ester derivative of sirolimus with enhanced pharmaceutical properties including improved stability and solubility and binds to a cytoplasmatic protein. This complex binds and inhibits mTOR and proved its potency in several clinical and laboratory studies [30]–[33].

We therefore evaluated the effect of temsirolimus on cellular events associated with retinal vascular diseases such as neovascular AMD and DR on retinal pigment epithelial cells and human umbilical vein endothelial cells as a common model for endothelial cells in vision research in an *in-vitro* model.

## Methods

### Ethics

The methods of securing human tissue were humane, complied with the Declaration of Helsinki, and were approved by the local ethics committee and institutional review board at Ludwig-Maximilians-University in Munich, Germany (file number AKIRB-20123). Samples were procured from our tissue bank („Bayerische Gewebebank Bavarian Tissue Banking GmbH“, http://www.klinikum.uni-muenchen.de/Augenklinik-und-Poliklinik/de/forschung-lehre/arbeitsgruppen/hornhautbank/index.html) and written informed consent was obtained before donor preparation from the donor or the next of kin.

### Materials

Temsirolimus((1*R*,2*R*,4*S*)-4-{(2*R*)-2-[(3*S*,6*R*,7*E*,9*R*,10*R*,12*R*,14*S*,15*E*,17*E*,19*E*, 21*S*,23*S*,26*R*,27*R*,34a*S*)-9,27-dihydroxy-10,21-dimethoxy-6,8,12,14,20,26-hexamethyl-1,5,11,28,29-pentaoxo 1,4,5,6,9,10,11,12,13,14,21,22,23,24,25, 26,27,28,29,31,32,33,34,34a-tetracosahydro-3*H*-23,27 epoxypyrido[2,1-*c*][1], [4] oxazacyclohentriacontin-3-yl]propyl}-2-methoxycyclohexyl 3-hydroxy-2-(hy-droxylmethyl)-2-methylpropanoate; LC Laboratories, Woburn, MA, USA) was dissolved in 100% dimethyl sulfoxide (DMSO; Sigma, St. Louis, MO, USA) and diluted with DMSO and the appropriate cell culture medium to the desired concentration with a final DMSO concentration of 0.1% for in vitro studies. DMSO was added to cultures at 0.1% (v/v) as a solvent control.

### Human RPE Cell Culture

Primary RPE cells from four human donors (aged 34, 47, 51, and 59 years old, obtained 3-10 h postmortem) without any history of eye disease were obtained from the Eye Bank of Ludwig Maximilian University and were prepared as previously described.[34] Dulbecco modified Eagle medium (DMEM; Biochrom, Berlin, Germany) supplemented with 10% fetal calf serum (FCS; Biochrom) was used as the cell culture medium. Primary human RPE cells of passage 3 to 7 were used for experiments.

### HUVEC Culture

Cultures of human umbilical vein endothelial cells (HUVEC) were purchased from Promocell (Heidelberg, Germany) and cultured according to the manufacturer’s instructions. Endothelial cell growth medium (ECGM; Promocell) with 5% FCS (Biochrom) was used as the cell culture medium. The experiments were conducted on HUVECs of passages 3 to 5.

### Treatment of Cell Cultures

For methylthiotetrazole (MTT; 3-[4,5-dimethylthiazol-2-yl]-2,5-diphenyl tetrazolium bromide) assays, cells were seeded in 6-well plates. For all other cell culture experiments, HUVECs or RPE cells were seeded in 35-mm tissue culture dishes and cultured on confluence in darkness. None of the used cell culture substrates were coated with anything aside of the polycarbonate membrane, that was coated with fibronectin and used in the boyden chamber assay.

For all experiments except proliferation and migration, before treatment, HUVECs or RPE cells were kept for 24 h in serum-free conditions. Then, for MTT testing regarding viability and proliferation, temsirolimus was added to reach final concentrations of 0.005, 0.05, 0.5, 1, 2.5, 5, 7.5, 10, 12.5, 15, 17.5, and 20 µg/mL.

As a result from viability testing and additional molecular pre-testing, temsirolimus at a final concentration of 0.05 µg/mL was chosen for all further experiments regarding growth factor expression.

### Hypoxic Stimulation

For hypoxia experiments, primary human RPE cells and HUVECs were grown to confluence. Human RPE cells were then washed and incubated overnight in serum-free medium. For hypoxic stimulation, dishes were placed in an incubator with 1% O_2_, 5% CO_2_, and 94% N_2_ in humidified atmosphere, for 24h. The controls were kept in humidified atmosphere of 5% CO_2_ in air at 37°C for the same time period.

### MTT Assay

The MTT assay was used to determine the cell survival rate. The MTT assay, which is well established for the assessment of cell viability, was performed as described by Mosmann, with some modifications.[34], [35] The medium was removed, the cells were washed with phosphate-buffered saline (PBS), and 1000 µL MTT solution (1.5 mL MTT stock, 2 mg/mL in PBS, plus 28.5 mL DMEM) was added to each well. RPE cells or HUVECs were incubated at 37°C for 1 h. The formazan crystals that formed were dissolved by the addition of 1000 µL DMSO per well. Absorption was measured by a scanning multiwell spectrophotometer at 550 nm (Molecular Probes, Eugene, OR, USA). The results are expressed as the mean percentage of proliferation in the control. The control was set to 100% to allow easier interpretation of the results.

### Evaluation of Cellular Viability

To investigate the effects of different concentrations of temsirolimus on viability of HUVECs and RPE cells, the cells were brought to confluence on uncoated well plates, kept under serum-free conditions for 24 h, and then treated with temsirolimus at concentrations 0.005, 0.05, 0.5, 1, 2.5, 5, 7.5, 10, 12.5, 15, 17.5, and 20 µg/mL for another 24 h. Then the MTT assay was performed. The control cells were HUVECs or RPE cells of the same passage.

### Cell Proliferation

To determine the effect of temsirolimus on cellular proliferation, HUVEC and RPE cells were seeded into a plain 6 well plate at a density of 3×10^4^ cells/well with medium containing 10% fetal calf serum. After four hours, final concentrations of 0.005, 0.05 and 0.5 µg/mL of temsirolimus and two controls were established in the culture plate. Cells were then incubated under standard cell culture conditions for 48 hrs. Then MTT solution (1.5 ml of 2 mg/ml in PBS plus 28.5 ml MEM) was added followed by 1 hour incubation at 37°C before 500 µl DMSO was added to dissolve the formazan crystals. Absorption was measured by a scanning multiwell spectrophotometer at 550 nm (Molecular Probes, Eugene, OR, USA). The control cells were HUVECs or RPE cells of the same passage. Controls were set to 100% to simplify reading of results.

### Migration Boyden Chamber Assay

Migration was observed by using microchemotaxis chambers (Neuroprobe, Gaithersburg, Maryland, USA) as described by Boyden with a few modifications.[36] The chamber consists of a lower and an upper part divided by a polycarbonate membrane (Nuclepore) with pores of 8 μm in diameter. In order to induce chemotaxis, 200 μl medium containing 1 ng/ml VEGF-165 (VEGF-165, Sigma-Aldrich, St Louis, Missouri, USA) was filled into the lower half of the chamber and covered with the polycarbonate membrane previously coated with fibronectin (coating time  =  24 hrs at 8°C and at a concentration of 2 μg/cm2). The upper half contained 1×10^5^ HUVEC or RPE cells cells in 750 μl of the according cell culture medium containing 5% FCS. Five chambers were prepared with three different concentrations of temsirolimus (0.005 µg/mL, 0.05 µg/mL, 0.5 µg/mL) and two controls containing the same amount of solvent. Cells were able to migrate for 24 hrs under standard cell culture conditions. Then all non-migrated cells on top of the membrane were removed by a cotton bud and the remaining cells on the bottom side were fixed with methanol for 10 mins. After fixation, a hematoxylin and eosin (HE) stain was used to make cells visible under a magnification of ×200 using an inverted phasecontrast microscope (Leica Microsystems GmbH, Solms, Germany). Cells of five representative areas were photographed using a digital camera (Leica, Solms, Germany) and manually counted using the Leica LAS particle counting tool. The assay was repeated four times on four different days. The results are presented as % control and controls are set to 100% in order to simplify result interpretation.

### RNA Isolation and Real-Time Polymerase Chain Reaction

Total RNA was isolated from HUVEC and RPE cells by the guanidinium thiocyanate-phenol-chloroform extraction method (Stratagene, Heidelberg, Germany) and quantification of VEGF (VEGF-A) and PDGF (PDGF-BB) mRNA was performed with specific primers using a LightCycler System (Roche Diagnostics, Mannheim, Germany) as described in our previous work [37]. Primers and probes were detected with ProbeFinder 2.04. Table 1 lists the primers used for RT-PCR. The level of VEGF and PDGF mRNA was determined as the relative ratio (RR), which was calculated by dividing the level of the respective mRNA by the level of the 18S rRNA housekeeping gene in the same samples. The ratios are expressed as decimals.

**Table 1:** Primers used for RT – PCR.

Target	Length	Position	AC (°C)	GC (%)	Sequence
VEGF - A	18	1540–1557	60	56	tgcccgctgctgtctaat
	18	1592–1609	60	61	tctccgctctgagcaagg
PDGF - BB	18	1108–1125	60	56	tgatctccaacgcctgct
	20	1156–1175	59	50	tcatgttcaggtccaactcg

### Protein Extraction and Western Blotting

HUVEC and RPE cells grown on 35-mm tissue culture dishes were washed twice with ice-cold PBS, collected, and lysed in RIPA cell lysis buffer. After centrifugation for 30 min at 19,000 × g in a cold microfuge (5810R; Eppendorf, Hamburg, Germany), the supernatant was transferred to fresh tubes and stored at –70°C for future use. The protein content was measured by the bicinchoninic acid protein assay (Pierce, Rockford, IL, USA). Denatured proteins (1e2 mg) were separated by electrophoresis under reducing conditions with the use of a 5% sodium dodecyl sulfate (SDS)-polyacrylamide stacking gel and a 12% SDS-polyacrylamide separating gel, transferred with semidry blotting onto a polyvinyl difluoride membrane (Roche), and probed with a mouse anti-VEGF-antibody or a mouse anti-PDGF-antibody (both (Santa Cruz Biotechnology Inc., Santa Cruz, CA, USA), as described previously [34]. Chemiluminescence was detected with an imager (LAS-1000; RayTest) and the generated light units. Exposure times ranged between 1 and 10 min. Quantification was performed on a computer (AIDA software; RayTest). Western blot lanes were manually labeled (“profile”) and analyzed by the one dimensional (1D) analysis mode of AIDA. Brightness was detected by the program and divided through the height of the profile. Resulting graphs were manually marked at the peak and the volume below calculated as the integral by the software. An even protein load in each lane was confirmed by staining of the polyvinyl difluoride membranes with Coomassie Brilliant Blue after the blotting procedure.

### Detection of VEGF and PDGF Secretion by HUVEC and RPE Cells

Cultures of HUVECs and RPE cells were grown to confluence and treated as described above. Levels of VEGF and PDGF in the culture supernatants were determined by enzyme-linked immunosorbent assays (ELISAs). The supernatants were collected after 24 h, and the levels of VEGF-A and PDGF were quantified using a VEGF (VEGF-A) or PDGF (PDGF-BB) Quantikine® ELISA Assay Kit (R & D Systems, Wiesbaden, Germany) according to the manufacturer’s instructions.

### Statistical Analyses

All data were analyzed with SPSS 13.0 for Windows (SPSS, Chicago, IL, USA). For all statistical tests, *P* < 0.05 was considered significant.

Results of the MTT assay for evaluating proliferation are presented as mean (SD) units of absorbance. Ten individual samples per group were measured in triplicate. For the toxicity study the results of the assay are presented as mean value displayed as bars with standard deviation compared to the control. Results from VEGF and PDGF ELISAs are presented as mean (SD) ratios of each tested probe, which were normalized to the control. Results of the RT-PCR are presented as mean (SD) ratios of the investigated mRNA and 18S rRNA. All experiments were performed at least in triplicate and repeated three times.

For statistical analyses a Wilcoxon test with α correction for multiple testing was used.

## Results

### Effects of Various Concentrations of Temsirolimus on Viability, Proliferation and Migration of HUVECs and Primary RPE Cells

#### HUVEC and RPE Cell Viability

**Figure 1:**
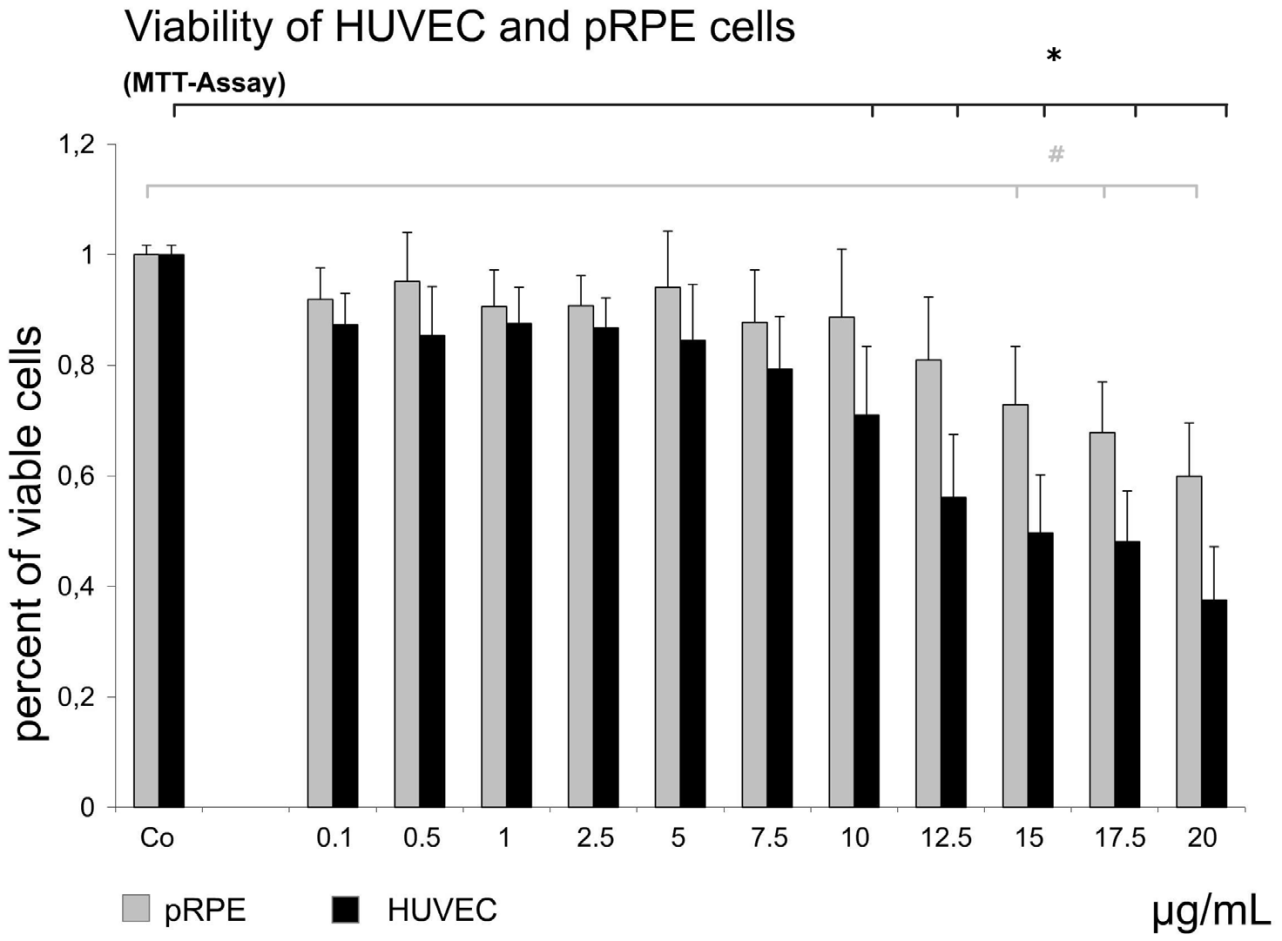
Viability of HUVEC and pRPE cells. Viability of HUVECs and primary human RPE cells after treatment with various concentrations of temsirolimus for 24 h, as measured by a colorimetric test (MTT). Untreated HUVECs or RPE cells of the same passage served as controls. Results are the mean percentages of control cell survival from three experiments, each carried out in triplicate, with error bars indicating SEM. Temsirolimus concentrations up to 12,5 µg/ml for RPE cells and 7,5 µg/mL for HUVEC showed no significant reduction in viability of either cell type (Wilcoxon test with a correction for multiple testing) compared to controls. Data are expressed as means ± SD. (*p<0.05 for HUVECs, #p<0.05 for RPE) Controls are set to 100% to simplify result reading.

To exclude that the detected molecular interactions of temsirolimus in HUVEC and RPE cells depend on toxic effects, viability of cells was investigated after exposure to temsirolimus concentrations. Results of the MTT assay showed no significant decrease in cellular viability in the cell cultures after 24 h of exposure to temsirolimus at concentrations between 0.005 and 7.5 µg/mL for HUVECs and 0.005 and 12.5 µg/mL for RPE cells (Fig. 1, Wilcoxon test with α correction for multiple testing). In HUVEC, concentrations of 10 µg/mL and higher showed an increasing reduction of cell viability. However, >60% of the cells still showed activity. It must be noted though, that an earlier study came to slightly different results: Zhang et al. investigated the effect of temsirolimus and found signs of toxicity to HUVECs at a concentration of 0.1 µg/mL.[38] They used this concentration since another group showed that sirolimus significantly inhibited proliferation in five different cell lines at this concentration. No toxicity was however appreciated in this study at the same concentration for these cells.[39], [40] Moreover is the intravenous concentration of temsirolimus (Torisel®, Pfizer, NY, USA) in the treatment of renal cell carcinoma significantly higher (30 mg). Therefore further studies are necessary to further illuminate this issue.

Beginning with a concentration of 12.5 µg/mL, cellular viability was reduced by >60% in a dose dependent manner. In RPE cells, concentrations beginning from 15 µg/mL temsirolimus led to a decrease of viability. However, even at the highest tested concentration of 20 µg/mL still >60% of cells were active (Fig. 1).

#### Proliferation of HUVEC and RPE Cells

**Figure 2:**
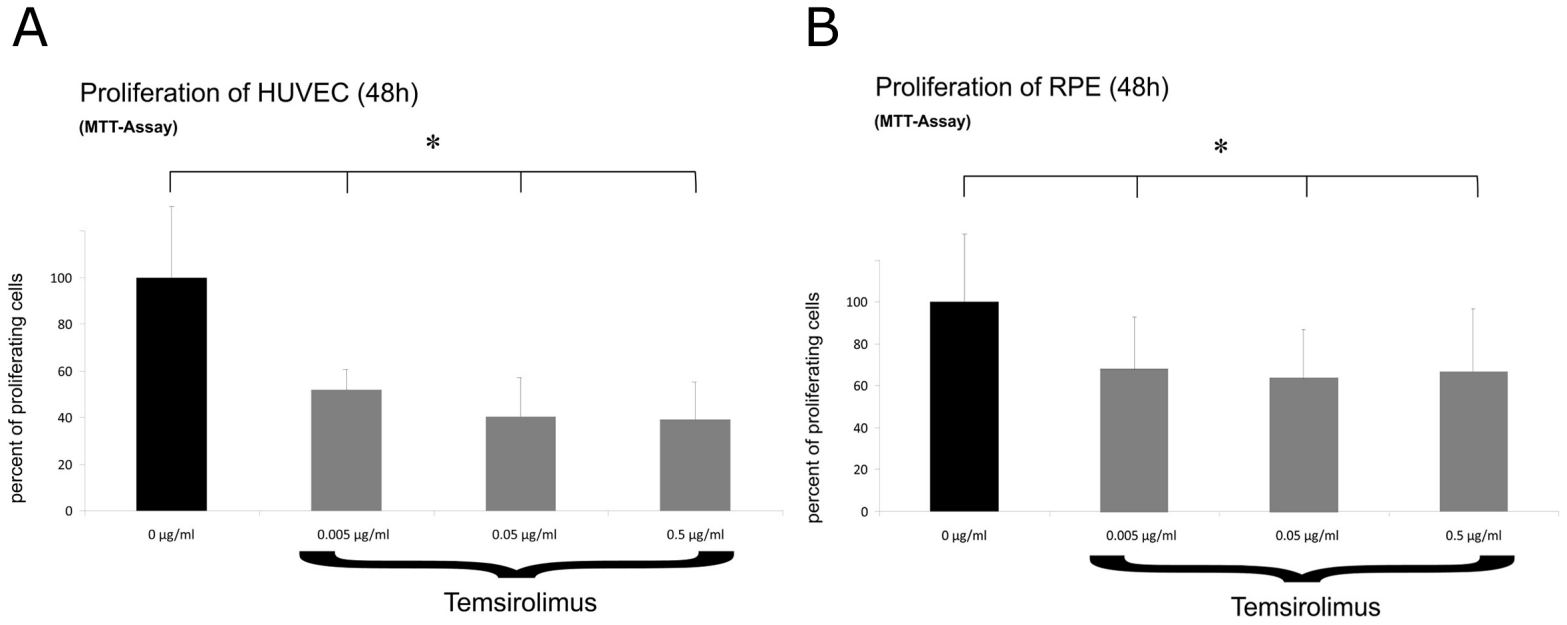
Proliferation of HUVEC and pRPE cells. Inhibition of HUVEC (2A) and RPE cell (2B) proliferation after incubation with three different concentrations of temsirolimus over 24 h measured by a colorimetric assay (MTT), (0 µg/mL  =  control). Data are expressed as means ± SD and results are displayed as % control, the control being set to 100%. (*p<0.05).

Cell proliferation is one of the key factors involved in neovascularisation. Therefore, we assessed HUVEC and RPE cell proliferation under different concentrations of temsirolimus (0.005, 0.05, and 0.5 µg/mL). At all tested temsirolimus concentrations (0.005, 0.05, and 0.5 µg/mL) and in both tested cell lines, HUVEC and RPE cells, a significant reduction in optical density was observed, compared to the untreated control (Wilcoxon Test, p<0.05) (Fig. 2A and 2B).

#### Migration of HUVEC and RPE Cells

**Figure 3:**
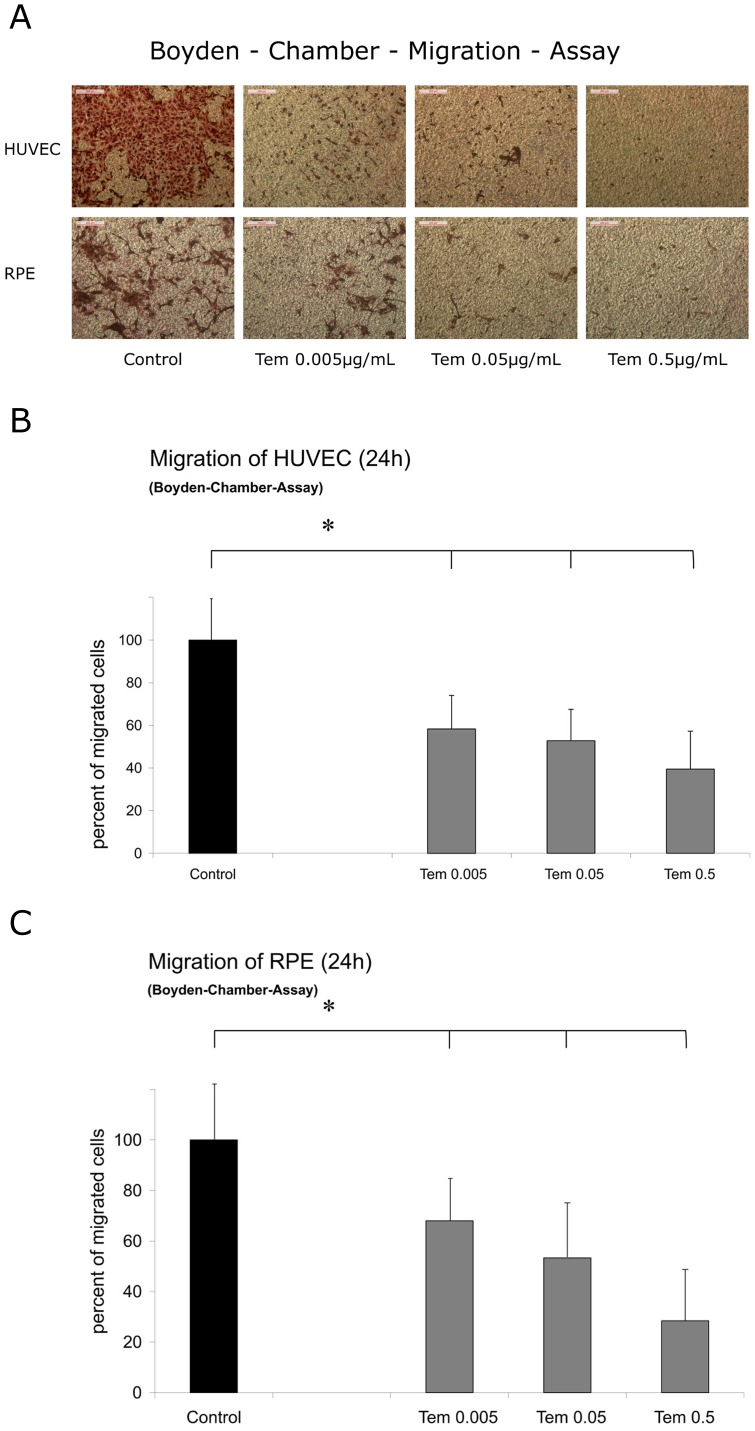
Migration of HUVEC and pRPE cells. HUVEC and RPE cell migration was significantly inhibited after exposure to temsirolimus in a dose-dependent manner (µg/mL) assessed by a modified Boyden chamber method. A) Example of cells attached to a permeable membrane at different concentrations compared to an untreated control. B) Evaluation of migrated HUVEC in percent control after treatment with three different temsirolimus concentrations. C) Migrated RPE cells compared to untreated cells in percent control. Data are expressed as means ± SD with *p<0.05.

The effects of temsirolimus on cellular migration were analyzed in vitro with a chemotactic approach. The chemotactic migration using VEGF-165, the isoform of VEGF that is most present in the eye, as an attractant was evaluated by using the Boyden Chamber. In both cell lines evaluated, HUVEC and RPE cells, there was a significant, dose dependent reduction in cell migration observed for temsirolimus concentrations (0.005, 0.05, and 0.5 µg/mL), compared to the control (Fig. 3A, 3B and 3C). Of note, there was also a significant difference in the decrease in-between the three tested temsirolimus concentrations (0.005, 0.05, and 0.5 µg/mL) with the strongest effect at the highest concentration of 0.5 µg/mL temsirolimus.

### Hypoxia Induced VEGF and PDGF Protein and mRNA Expression in HUVECs and RPE Cells

#### Expression of VEGF and PDGF mRNA

**Figure 4:**
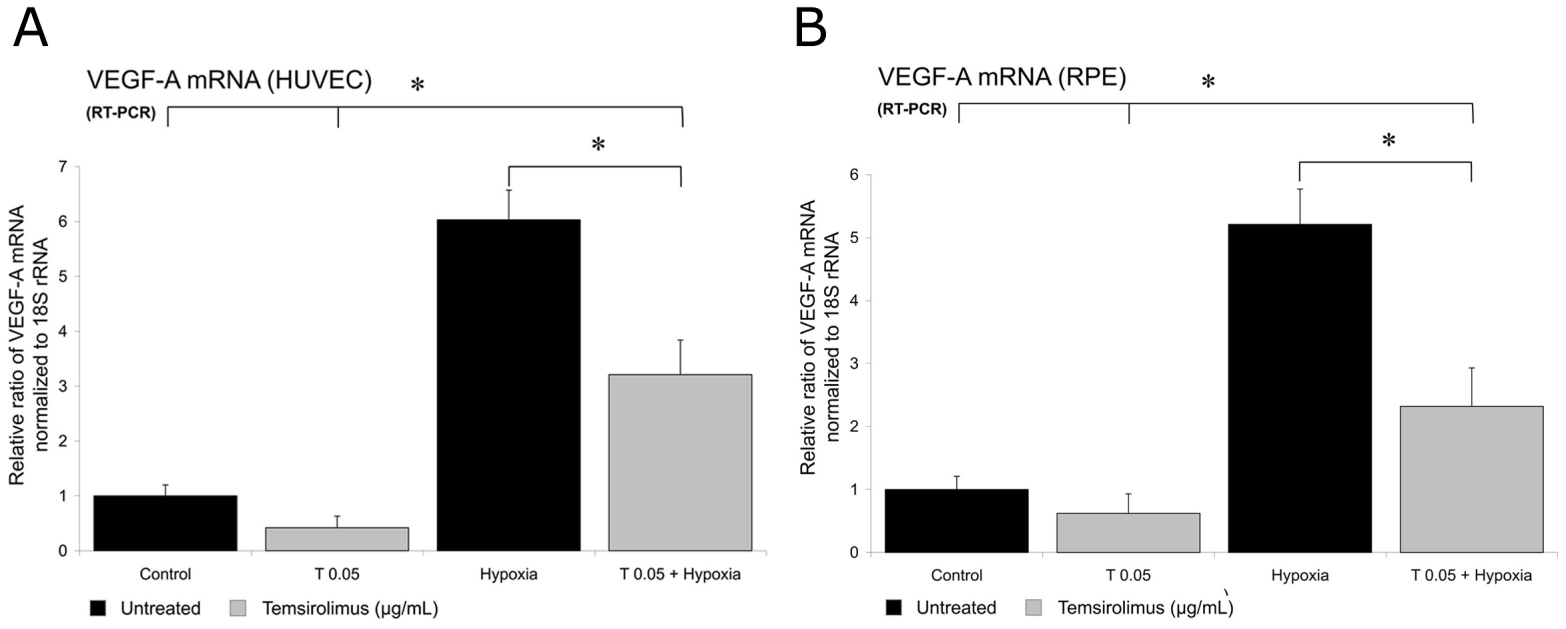
VEGF mRNA expression in HUVEC and pRPE cells. VEGF (VEGF-A) expression of untreated HUVECs and primary RPE cells and after exposure to hypoxia for 24 h, as measured by quantitative RT-PCR. When cells were exposed to hypoxia, a significant increase in VEGF mRNA could be detected in both tested cell lines. When cells were additionally treated with 0.05 µg/ml temsirolimus, in HUVEC (A) and RPE cells (B) the hypoxia-induced increase in VEGF mRNA expression was significantly less. Y-axis: RR of VEGF mRNA normalized to 18S rRNA, expressed in decimal format. (*p<0.05).

**Figure 5:**
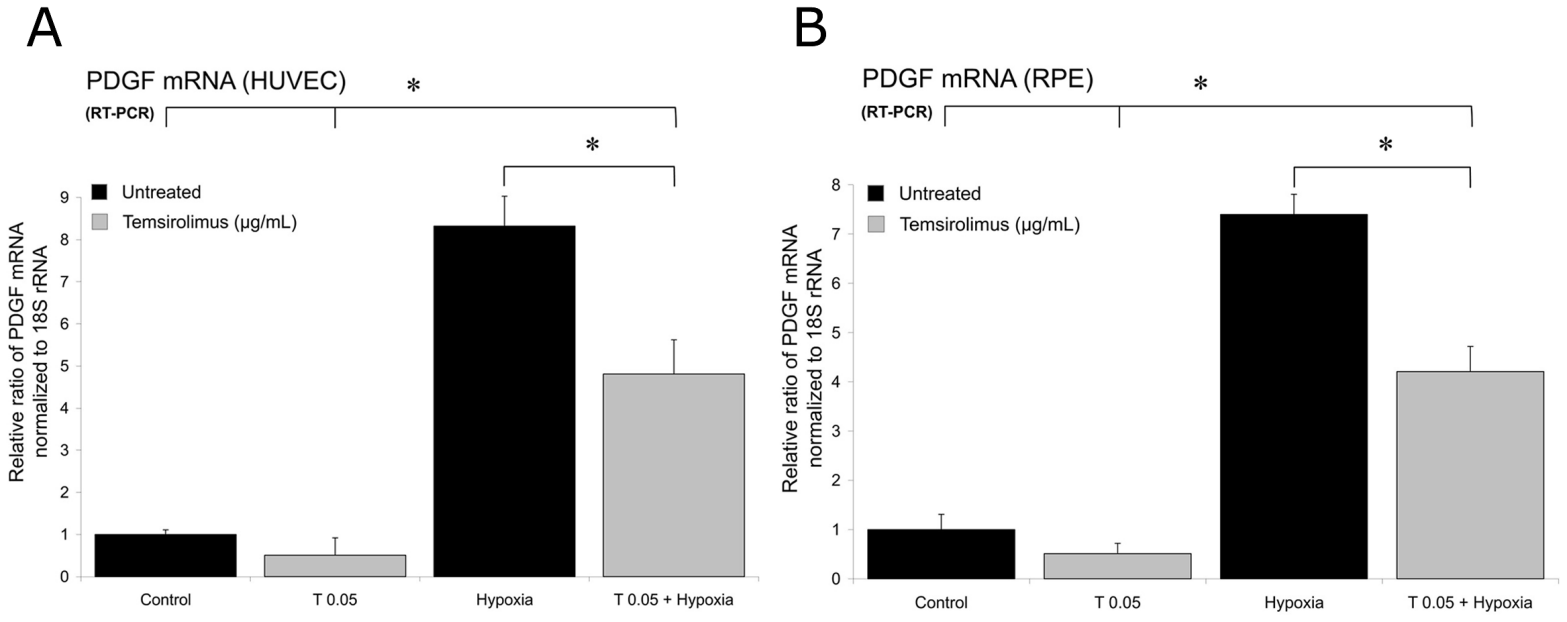
PDGF mRNA expression in HUVEC and pRPE cells. PDGF (PDGF-BB) expression of untreated HUVECs (A) and primary RPE cells (B) and after exposure to hypoxia for 24 h, as measured by quantitative RT-PCR. Experiments were carried out in a similar manner as described in Figure 4. After hypoxia, additional treatment with 0.05 µg/mL temsirolimus led to a significantly less increase of PDGF expression. Y-axis: RR of PDGF mRNA normalized to 18S rRNA, expressed in decimal format. (*p<0.05).

Exposure to hypoxia for 24 h led to a significant increase in VEGF and PDGF mRNA expression in both HUVECs and RPE cells. Treatment of both cell lines with 0.05 µg/mL temsirolimus significantly reduced this hypoxia-induced increase in VEGF (Fig. 4A and 4B) and PDGF mRNA (Fig. 5A and 5B) after 24 h.

### Expression of VEGF and PDGF Protein

#### Western-Blotting from Cell Lysates

**Figure 6:**
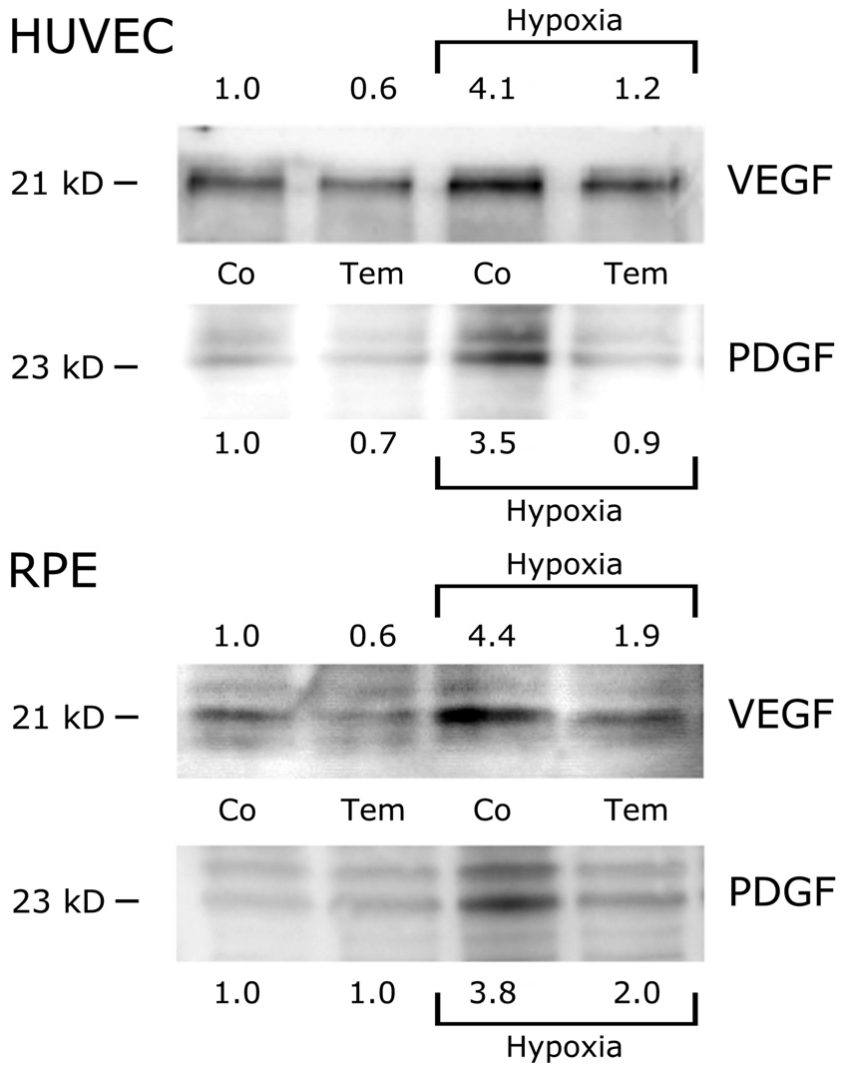
Protein levels of VEGF and PDGF in HUVEC and pRPE cells by Western Blot analysis. Representative Western blots showing the expression of VEGF (VEGF-A) and PDGF (PDGF-BB) in untreated HUVEC and RPE cells (Co) and treated with 0.05 µg/ml of temsirolimus (Tem) after 24 h exposure to normoxic conditions or hypoxia. Ten micrograms of protein were loaded per lane. An even protein load in each lane was confirmed by staining of the polyvinyl difluoride membranes with Coomassie Brilliant Blue after the blotting procedure.

For verification that the hypoxia-induced decrease of VEGF and PDGF in mRNA transcription translates into increased protein synthesis, whole cellular protein extracts of untreated control cells, cells exposed to hypoxia without temsirolimus treatment, and cells treated with 0.05 µg/mL temsirolimus and exposure to hypoxia were analysed by western blotting. Under hypoxic conditions, a marked increase in both VEGF and PDGF expression could be detected compared with the control. In HUVECs a 4.1x and 3.5x increase could be appreciated for VEGF and PDGF respectively, while for RPE this increase was even a little bit stronger with 4.4x for VEGF and 3.8x for PDGF compared to the control. When HUVEC and RPE cells were treated with 0.05 µg/mL temsirolimus and then exposed to hypoxia however, expression of both VEGF and PDGF protein was noticeably lower than after exposure to hypoxia only. In the presence of temsirolimus the increase of VEGF and PDGF was reduced to 1.2x and 0.9x in HUVECS and 1.9x and 2.0x in respect of RPEs (Figure 6).

#### Detection of VEGF and PDGF Secretion in HUVECs and RPE Cells

**Figure 7:**
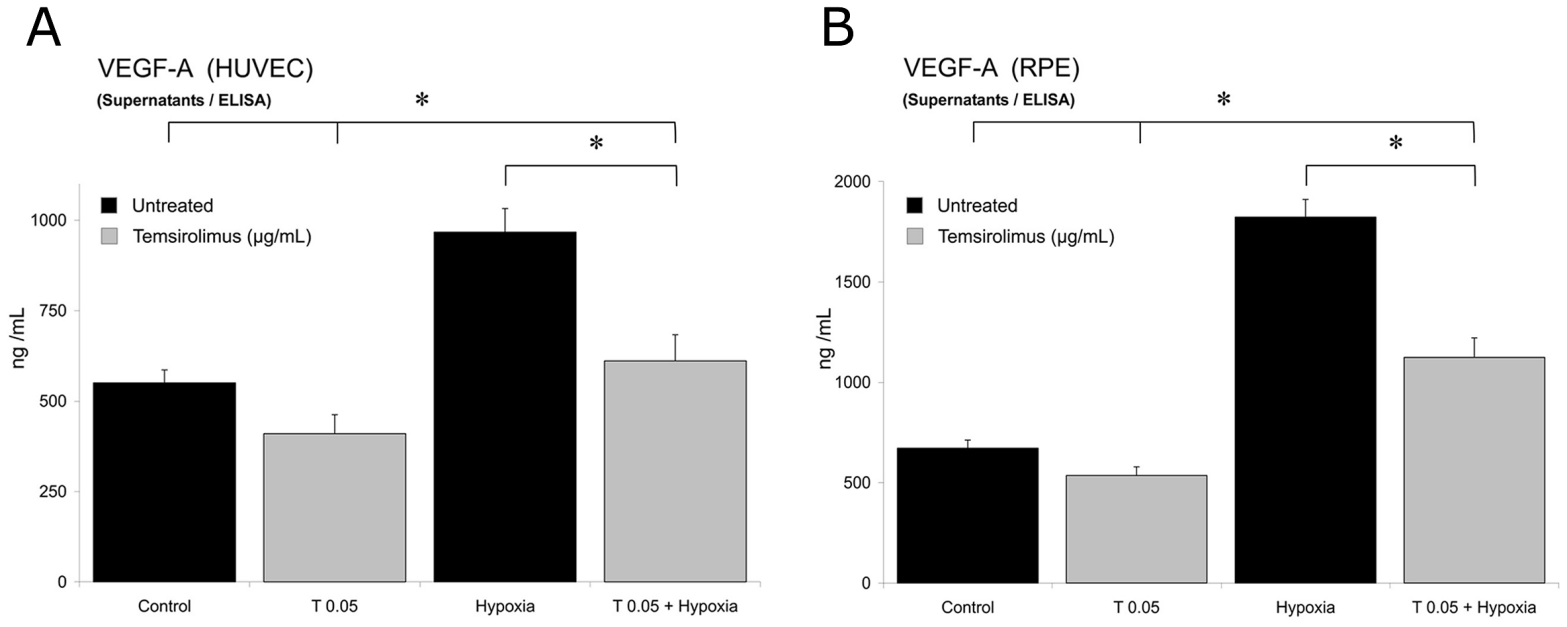
Protein levels of VEGF in HUVEC and pRPE cell supernatants (ELISA). Inhibitory effect of temsirolimus on VEGF expression, as measured by ELISA from supernatants. After exposure to 0.05 µg/ml temsirolimus for 24 h, a significant decrease in VEGF protein expression of both HUVEC (A) and RPE cells (B) was detected. Each value was normalized to a standard curve of VEGF, and normalized to the untreated control of each cell line. Data are expressed as means ± SD. (*p<0.05).

**Figure 8:**
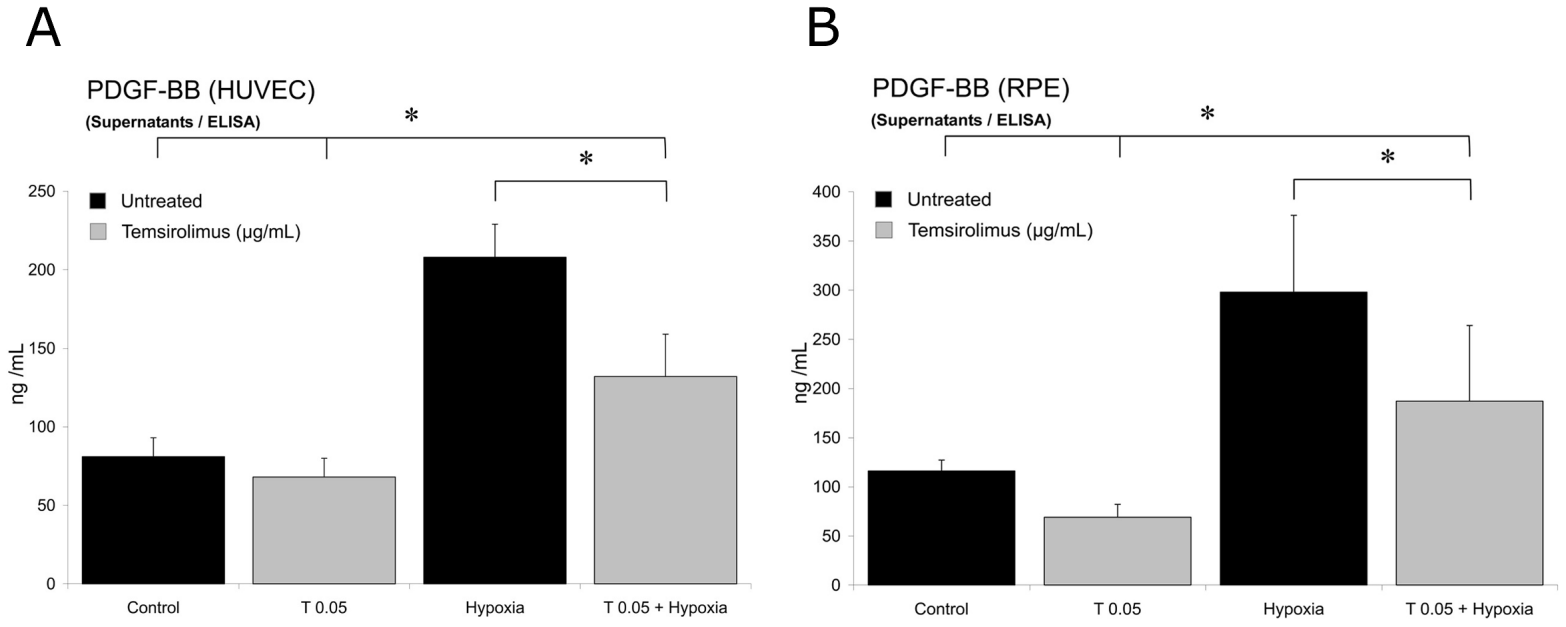
Protein levels of PDGF in HUVEC and pRPE cell supernatants (ELISA). Protein levels of PDGF were measured in a similar manner as VEGF expression from supernatants with an ELISA assay. Cells were exposed to 0.05 µg/ml temsirolimus for 24 h. A significant decrease in PDGF protein expression for both HUVEC (A) and RPE cells (B) was detected. Each value was normalized to a standard curve of PDGF, and normalized to the untreated control of each cell line. Data are expressed as means ± SD. (*p<0.05).

To investigate the effect of temsirolimus on VEGF and PDGF (PDGF-BB) secretion after exposure to hypoxia, levels of VEGF (Fig. 7A and 7B) and PDGF (Fig. 8A and 8B) in cell culture supernatants were quantified using ELISA after 24 h. A significant increase of the secretion of VEGF and PDGF by cultured HUVECs and RPE cells was noted after 24 h of exposure to hypoxia. Treatment of either cell line with temsirolimus 0.05 µg/mL reduced the secretion of VEGF and PDGF significantly compared to controls.

## Discussion

Without any doubt, intravitreal inhibition of VEGF revolutionized the therapy of a number of retinal vascular diseases [41], [42]. The selective inhibition of VEGF and its isoforms is one of the major strength of this treatment approach as it goes along with only very few side effects [43], it is however conceivable that a broader therapeutic approach involving the inhibition of key signal pathways might be of interest especially for those patients, who do not sufficiently benefit from the inhibition of a single molecule such as VEGF [44].

The mammalian target of rapamycin (mTOR) is one of the key signaling complexes that had been identified in conditions that are linked with angiogenic features such as increased cellular proliferation and migration [45] and evidence has been provided that mTOR inhibition could be a suitable therapeutic strategy for neovascular, degenerative retinal diseases [46].

We therefore investigated whether temsirolimus, a rapalog (rapamycin analog), would have inhibitory features on RPE and human umbilical vein endothelial cells (HUVEC) in non-toxic concentrations in an *in-vitro* setting. As a first important result of our experiments we could clearly show that temsirolimus concentrations between 0.005 µg/mL and 7.5 µg/mL for HUVECs and 0.005 µg/mL and 12.5 µg/mL for RPE cells showed no decreased viability (Figure 1). Always in mind that these results are only *in vitro*, our data give a clear hint towards a broad therapeutic range of the substance and that the concentrations used for our further experiments (0.005 µg/mL – 0.5 µg/mL) can be estimated as safe and non-toxic. MTOR consists of two protein complexes: mTOR complex 1 and 2 (mTORC 1 & 2). Once a growth factor such as VEGF binds to its receptor, particularly VEGFR-2, a signaling cascade is started leading to activation of PI3K and subsequent activation of protein kinase B (Akt) [29]. The effect of this is a regulation of mTORC 1 and a consecutive up regulation of eukaryotic initiation factor 4E-binding protein (4E-BP1) and the ribosomal protein S6 kinase (S6K) [47]. The phosphorylation of these proteins eventually leads to increased levels of several proteins that are important in cellular proliferation, migration and angiogenesis in RPE and vascular endothelial cells [48]–[52], being crucial cellular features in the pathogenesis of retinal vascular diseases such as neovascular AMD or proliferative DR [53]. In our experiments, a significant dose dependent effect on RPE cells as well as on HUVECs was seen in terms of inhibiting proliferation (Figure 2) as well as migration (Figure 3) after treatment with three different concentrations of temsirolimus, suggesting that a significant reduction of these properties can be reached by directly targeting mTOR without specifically blocking VEGF or other involved cytokines. However, in previous investigations using cancer cell lines, the inhibition of mTOR not only showed reduced activation in respect of cell proliferation and migration but also decreased activity of PI3K/Akt itself, suggesting susceptibility of even upstream mediators [54], [55]. The pathogenesis of ME in retinal vascular disease is multifactorial and mainly results from a breakdown in the blood–retina barrier (BRB) separating the neurosensory retina from the vascular components of the eye. A disruption of the BRB involves an abnormal inflow of fluid into the neurosensory tissue that often causes residual accumulation of fluid in the intraretinal layers of the macula [56].

It is well known, that hypoxia is one of the leading causes for increased expression of VEGF, including its isoforms leading to the breakdown of the BRB [5], [57]. VEGF is expressed by all vascularized retinal tissues and there is clear evidence that in response to hypoxia, augmented expression of VEGF in retinal pericytes, RPE and endothelial cells occurs [58], [59].

We therefore induced elevated VEGF and PDGF levels in both RPE and endothelial cells by exposing them to hypoxic conditions and analyzed a possible effect of temsirolimus in respect of VEGF and PDGF levels on RNA and protein levels. Our results implicate that a significant reduction of VEGF and PDGF levels can be achieved by intervening at the level of mTOR, a downstream mediator of VEGFR-2 and VEGF, by temsirolimus following increased growth factor expression by induction of hypoxia in both cell types (Figure 4, 5 and Table S1). We furthermore proved that this reduction eventually lead to a decrease in protein levels for both cytokines qualitatively (Figure 6) as well as quantitatively (Figure 7).

Mizukami et al. previously postulated however, that not only hypoxia, by stabilization of HIF-1, cellular receptors such as EGFR and IGF-IR and other growth factors (e.g. PDGF) induce the production of VEGF and its receptors but also PI3K and its downstream signaling pathway compounds including mTOR itself might trigger their production [60]–[64]. The strong decrease of VEGF expression in our experiments might therefore also emphasize the idea of mTOR being directly involved in the expression of VEGF and other growth factors.

Our experiments clearly show that temsirolimus has a broad therapeutic range and even low concentrations were capable of inhibiting proliferation and migration significantly in RPE and endothelial cells. Our results additionally suggest that temsirolimus is able to directly decrease the expression of growth factors such as VEGF and PDGF both on RNA and protein levels.

These findings are particularly interesting as the combination of an anti-VEGF drug and temsirolimus might have complimentary therapeutic effects for patients who do not benefit from repeated intravitreal anti-VEGF injections. This combination however, has not been studied extensively and therefore no valid statement regarding synergistic effects with anti-VEGF drugs can be made. Due to the very low concentrations that are needed to achieve significant effects and currently ongoing studies to evaluate the intravitreal biocompatibility of sirolimus [65], another mTOR inhibitor of the same family, we believe that toxic side effects described, when taken orally in order to treat different cancers would be less likely [66].

Temsirolimus is a well-tolerated and approved drug for renal cell carcinoma [67]. Our results show first evidence that inhibiting mTOR, a central signaling pathway in the complex cascade underlying neovascularization and angiogenesis, with temsirolimus lead to a decrease in the production of the key cytokine VEGF as well as other growth factors such as PDGF and also reduce cellular events typically associated with angiogenesis.

Our very early results on the effect of mTOR inhibition in this context are promising, although they are solely *in-vitro* with typical limitations including investigating mono cell cultures and cells that are not in the proper physiological state. (Figure S1)

We nonetheless believe, that temsirolimus might have a role in the treatment of ocular neovascular diseases in the future and further investigations should be pursued to gain additional information regarding clinical outcome as well as biocompatibility.

## Supporting Information

Figure S1 Typical limitations of mono cell culture based experiments include cells that are not in the proper physiological state. Photographs of both human umbilical vein endothelial cells (HUVEC) [A] and primary retinal pigment epithelial cells (RPE) [B] that were used for our experiments are shown at near confluence.
(JPG)

Table S1 Results for normalized ratios of rtPCR using RelQuant 1.01 (Roche Diagnostics, Mannheim, Germany) in mono color mode.
(DOCX)
