# Supplementary figures and images for: Temsirolimus Inhibits Proliferation and Migration in Retinal Pigment Epithelial and Endothelial Cells via mTOR Inhibition and Decreases VEGF and PDGF Expression
Source: PLoS One. 2014 Feb 26;9(2):e88203. doi: 10.1371/journal.pone.0088203 (PMC3935828; doi:10.1371/journal.pone.0088203)

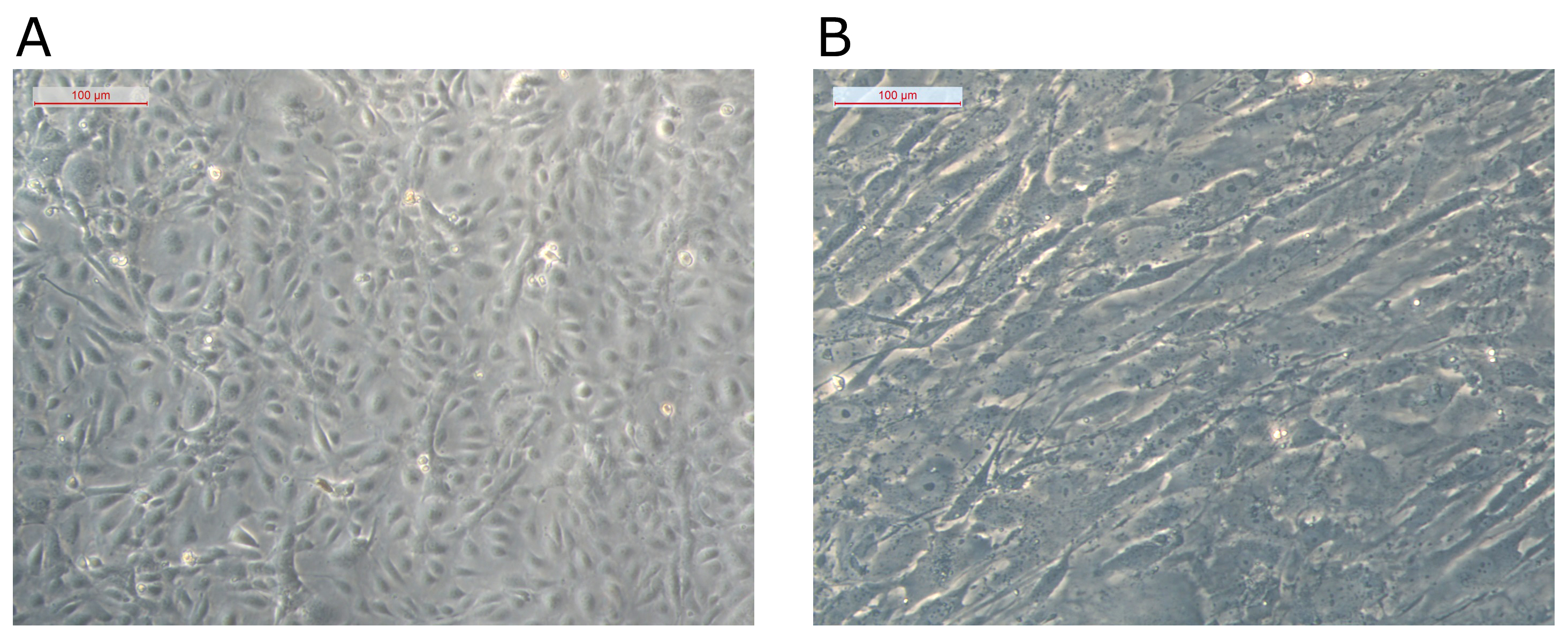

Supplement: Figure S1 — Typical limitations of mono cell culture based experiments include cells that are not in the proper physiological state. Photographs of both human umbilical vein endothelial cells (HUVEC) [A] and primary retinal pigment epithelial cells (RPE) [B] that were used for our experiments are shown at near confluence. (JPG) [file pone.0088203.s001.jpg]
