# Supplementary material for: Temsirolimus Inhibits Proliferation and Migration in Retinal Pigment Epithelial and Endothelial Cells via mTOR Inhibition and Decreases VEGF and PDGF Expression
Source: PLoS One. 2014 Feb 26;9(2):e88203. doi: 10.1371/journal.pone.0088203 (PMC3935828; doi:10.1371/journal.pone.0088203)
Supplement: Table S1 — Results for normalized ratios of rtPCR using RelQuant 1.01 (Roche Diagnostics, Mannheim, Germany) in mono color mode. (DOCX) [file pone.0088203.s002.docx]

|  | **VEGF** | | **PDGF** | |  |
| --- | --- | --- | --- | --- | --- |
|  | pRPE | HUVEC | pRPE | HUVEC |  |
|  | Control | 1.00 ± 0.21 | 1.00 ± 0.22 | 1.00 ± 0.31 | 1.00 ± 0.09 |
|  | Temsi  0,05µg/mL | 0.62 ± 0.3 | 0.41 ± 0.21 | 0.61 ± 0.19 | 0.51 ± 0.39 |
| Hypoxia | Control | 5.29 ± 0.57 | 6.07 ± 0.53 | 7.54 ± 0.39 | 8.32 ± 0.70 |
|  | Temsi  0,05µg/mL | 2.35 ± 0.61 | 3.22 ± 0.64 | 4.25 ± 0.52 | 4.78 ± 0.79 |
